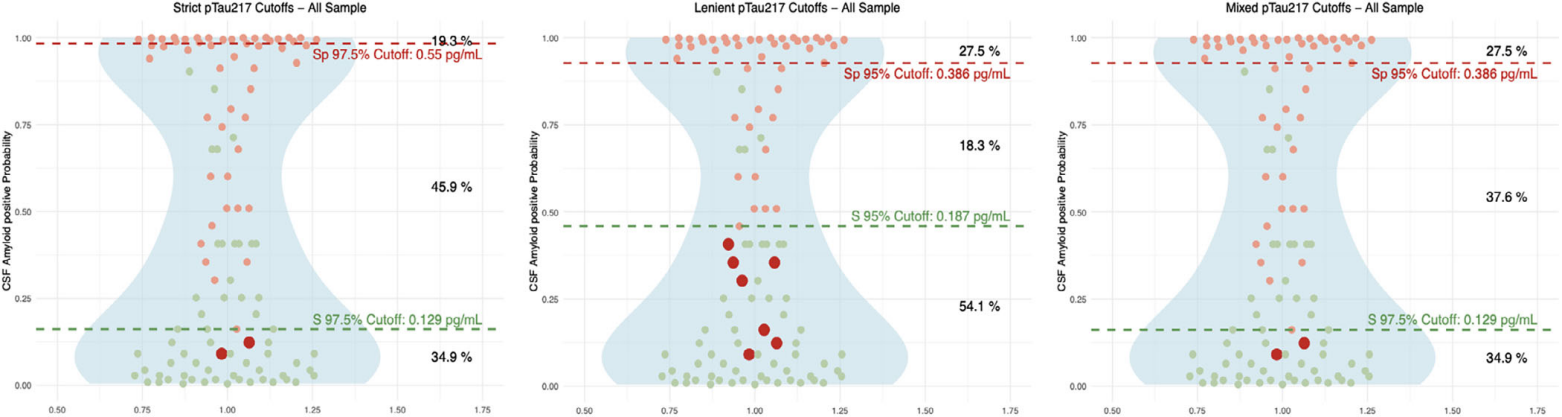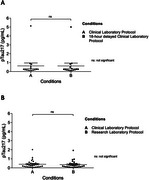# Prospective evaluation of plasma pTau_217_ stability and diagnostic accuracy for the detection of Alzheimer's disease in a memory clinic

**DOI:** 10.1002/alz70856_105569

**Published:** 2026-01-07

**Authors:** Javier Arranz, Rosa Ferrer, Nuole Zhu, Sara Rubio‐Guerra, Íñigo Rodríguez‐Baz, José Enrique Arriola‐Infante, Lucía Maure‐Blesa, Jesús Garcia Castro, Maria Carmona‐Iragui, Isabel Barroeta, Ignacio Illán‐Gala, Miguel A Santos‐Santos, Juan Fortea, Alberto Lleó, Mireia Tondo, Daniel Alcolea

**Affiliations:** ^1^ Sant Pau Memory Unit, Hospital de la Santa Creu i Sant Pau, Institut de Recerca Sant Pau ‐ Universitat Autònoma de Barcelona, Barcelona, Spain; ^2^ Barcelona Down Medical Center, Sant Pau Hospital, IR SANT PAU, Fundació Catalana Síndrome de Down, Barcelona, Barcelona, Spain; ^3^ Sant Pau Hospital, IR SANT PAU, Universitat Autònoma de Barcelona, Barcelona, Barcelona, Spain; ^4^ CIBERNED, Network Center for Biomedical Research in Neurodegenerative Diseases, National Institute of Health Carlos III, Madrid, Spain; ^5^ Sant Pau Memory Unit, Hospital de la Santa Creu i Sant Pau ‐ Biomedical Research Institute Sant Pau ‐ Universitat Autònoma de Barcelona, Barcelona, Barcelona, Spain; ^6^ Sant Pau Memory Unit, Hospital de la Santa Creu i Sant Pau ‐ Biomedical Research Institute Sant Pau ‐ Autonomous University of Barcelona, Barcelona, Catalonia, Spain; ^7^ Barcelona Down Medical Center, Fundació Catalana Síndrome de Down, Barcelona, Spain; ^8^ Sant Pau Memory Unit, Department of Neurology, Institut d’Investigacions Biomèdiques Sant Pau‐Hospital de Sant Pau, Universitat Autònoma de Barcelona, Barcelona, Barcelona, Spain; ^9^ Unidad Alzheimer‐Down, Department of Neurology, IR SANT PAU, Hospital de la Santa Creu i Sant Pau; Barcelona Down Medical Center, Fundació Catalana Síndrome de Down., Barcelona, Barcelona, Spain; ^10^ Center for Biomedical Investigation Network for Neurodegenerative Diseases (CIBERNED), Madrid, Spain; ^11^ Center for Biomedical Investigation Network for Neurodegenerative Diseases (CIBERNED), Madrid, Madrid, Spain; ^12^ Centro de Investigación Biomédica en Red en Diabetes y Enfermedades Metabólicas, CIBERDEM, Barcelona, Barcelona, Spain; ^13^ Institut d’Investigacions Biomèdiques Sant Pau ‐ Hospital de Sant Pau, Universitat Autònoma de Barcelona, Servei de Bioquímica, Barcelona, Spain

## Abstract

**Background:**

Knowledge on the effect of analytical variability and storage conditions are essential for the successful implementation of plasma pTau_217_ in prospective settings. The aim of this study is to investigate the diagnostic performance of plasma pTau_217_, measured with LUMIPULSE, for detecting Alzheimer's disease in a prospective memory clinic setting, along with evaluating its pre‐analytical and analytical stability.

**Method:**

We prospectively measured pTau_217_ using the LUMIPULSE automated platform in consecutive patient samples collected between May and November 2024 at the Sant Pau Memory Unit (Barcelona). A subset of participants underwent lumbar puncture for CSF AD biomarkers. We compared biomarker concentrations under different short‐term storage conditions (4°C vs ‐20°C) and assessed lot‐to‐lot variability. In the subset with available CSF biomarkers, logistic regression was used to evaluate the association between plasma pTau217 and the likelihood of a positive (A+) or a negative (A‐) CSF amyloid status. Using ROC analysis, in this prospective cohort we evaluated the accuracy of previously established thresholds derived from historical samples.

**Result:**

We included 280 participants, divided into two groups: those with CSF data (*n* = 109) and those without CSF data (*n* = 171). Among the subset with CSF, 48% were A+, with a plasma pTau_217_ fold‐change of 4.5x compared to A‐. We found no differences in plasma pTau_217_ concentrations between both short‐term storage conditions. The overall coefficients of analytical variation ranged from 1.8% to 3.2%. Following a two‐threshold approach, the need of confirmatory tests (grey zones) after measuring plasma pTau_217_ ranged between 45.9% using our previously reported strict cutoffs (overall accuracy 96.6%) and 18.3% using our previously reported lenient cutoffs (overall accuracy 92.1%).

**Conclusion:**

The robust stability and low lot‐to‐lot variability of plasma pTau_217_ measurement in an automated plaftorm result in high diagnostic performance of this biomarker in the prospective evaluation of patients in a memory clinic setting. These findings support its implementation into clinical routine, offering clinicians an accessible biomarker for AD diagnosis.